# Defining Moral Injury: A Natural Scaffold

**DOI:** 10.1007/s10943-026-02626-1

**Published:** 2026-03-27

**Authors:** David G. S. Smith

**Affiliations:** Birmingham City University, Birmingham, UK

**Keywords:** Moral injury, Natural justice, Natural law, Moral conscience, Ethics, Moral philosophy

## Abstract

The concept of moral injury (MI), while increasingly referenced across disciplines, remains under-theorised philosophically, with current definitions often relying on subjective intuitions of “what is right.” This paper addresses a critical gap by interrogating the ethical foundations of MI and proposing a normative framework that resists conceptual drift. Drawing on natural law theory with its roots in natural justice, I argue that the developing construct requires grounding in principles of intelligibility, universality, and objectivity—all criteria essential for any robust ethical theory. Through a systematic conceptual analysis, I delineate the dynamic interplay between *ethics*, *moral conscience*, and *behaviour*, demonstrating how natural law thinking can safeguard the construct from moral relativism and ideological annexation. This approach not only strengthens definitional clarity but also enhances interdisciplinary applicability, offering a philosophically rigorous foundation for future research and practice. By anchoring MI within an enduring ethical tradition, the paper advances its theoretical development and positions it as a concept of significant interdisciplinary and societal relevance.

## Introduction

At the time of writing, the concept of moral injury (MI) remains in its developing stage, with no commonly agreed definition available. MI was first described using a literary/narrative approach by the American military psychiatrist Jonathan Shay when he reflected that [Vietnam] “veterans usually recover from horror, fear, and grief once they return to civilian life, so long as “what’s right” has not also been violated” (Shay, 1994 p. 19). Shay’s initial description of MI later crystalised into a more formal working definition of MI in his 2014 paper as “a betrayal of what’s right, by someone who holds legitimate authority (e.g. in the military—a leader) in a high-stakes situation. All three” (Shay, 2014 p. 183). In the intervening period, other psychologists were developing provisional definitions of MI that sought to explore the phenomenon holistically. In a milestone paper, Brett Litz’s team formulated MI as “perpetrating, failing to prevent, or bearing witness to acts that transgress deeply held moral beliefs and expectations may be deleterious in the long term, emotionally, psychologically, behaviourally, spiritually, and socially" (Litz et al., 2009 p. 695).

Initial working definitions of MI (above) have been adapted and expanded thematically by later writers who added context and varying degrees of precision to the project (Atuel et al., 2021; Carey & Hodgson, 2018; Jinkerson, 2016; Koenig et al., 2019; Litz & Walker, 2025; Litz et al., 2022; VanderWeele et al., 2025). Initial military-focused definitions have been enlarged by thematic insights of other disciplines such as healthcare and education (ter Heide & Ollf, 2023; Levinson, 2015; Williamson et al., 2020).

Much of the academic literature comments on the holistic effects of MI on those affected, exploring repair and healing pathways (Antal, 2023; Antal et al., 2022; Atuel et al., 2020; Carey et al., 2023). There is, however, a paucity of philosophical scrutiny of the ethical and moral foundations on which the MI construct rests, although some writers have addressed these tangentially. Atuel et al. (2021), for example, use an Aristotelian virtue approach to present the non-clinical element of a dual pathway model of understanding moral character formation and MI. Fiala (2017) explores how qualities of *moral seriousness* and *moral sensitivity* in combatants may increase susceptibility to MI if the moral case for going to war (*jus ad bellum*) is unconvincingly made or if underlying requirements of justice are poorly understood.^1^ Jeannine Suurmond (2026) uses a literary approach, similar to Shay’s, to highlight and connect transferable moral themes associated with MI in the Mediaeval epic poetry of Dante’s (1265–1321)* Divine Comedy.*

### Socratic Aporia

This paper highlights a Socratic *aporia* or developmental pause that seeks to identify, interrogate and connect some of the theoretical building blocks of the MI concept. My intention is to explore—and to some extent deconstruct—the ethical foundations of MI theory, seeking to offer a sharper philosophical framework for understanding the biological–psychological–social–spiritual deleterious effects on the human person arising from a violated moral conscience (Boudreau, 2011; Carey et al., 2023; Hynes et al., 2025; Koenig & Al Zaben, 2021). My starting point is rooted not in psychology or clinical practice (though this exploration may have implications for them) but in the domain of the human person understood as a moral actor. It is not my intention in this paper to construct a grand theory or to give another definition of MI – rather I seek to offer some complementary building blocks and conceptual perspectives that researchers working within an interdisciplinary approach to MI will find fruitful as the project develops. The methodology and theoretical assumptions I work within and advance in this exploration are qualitative in character.

Unpacking Shay’s original connection between Greek ideas of *Themis* and MI, I will argue that for the term “moral” to cohere philosophically, it requires reference to an agreed external or meta-ethical framework of “what is right” that necessarily harnesses the moral conscience or the capacity for making a judgement of practical reason, native to the human person. Future definitions of MI, I argue, should articulate a clearer dynamic link between key conceptual building blocks: natural justice; natural law; ethics; moral conscience; and moral behaviours. In this way, this paper seeks to offer a novel philosophical scaffold for the emerging MI construct that is rooted firmly within the holistic moral domain of the human person.

### Natural Thinking

Natural law ethical thinking, grounded in ideas of natural justice and its virtuous outworkings, I argue, offers an approach for apprehending human behaviours that makes it uniquely suited to account for themes of transgression, guilt, and shame that form core manifestations of MI. Unlike rival ethical theories, natural law ethical theory claims the unique ability to transcend cultural, political, and power causal reasoning by rooting its thinking in ideas of natural justice discernible by native human rationality reflecting, over time, on the nature of things. In this respect, natural law thinking can claim universality in ethical matters since, notwithstanding cultural variety of expression, all human societies depend upon a small number of commonly agreed values or ethical norms without which societies could not function. At its most basic, as sociologist Clyde Kluckhohn notes, “every culture has a concept of murder, distinguishing this from execution, killing in war and other ‘justifiable homicides’”. This, and other moral concepts, he notes, “are altogether universal” (Kluckhohn, 1955 p. 663–677). In his study of the morally collapsing Ik tribe in Uganda, the anthropologist Colin Turnbull noted the inevitability of social disintegration and barbarism that flowed from a people without shared concepts of kindness, loyalty, and collaboration (Turnbull, 1972).

Competing ethical theories such as Divine Command Theory, Kantian Duty Ethics (Deontology) and Utilitarianism, while commenting on some aspects of human nature and behaviour, fail to account systematically and convincingly for it. Ethical Utilitarian theories such as Consequentialism, for example, rely ultimately on questions of power and authority to compel behaviours that are situationally ordered towards the greatest happiness for the greatest number with the minimisation of pain. The moral worth of an action is thereby evaluated by weighing its potential consequences in the light of this fundamental premise. In Utilitarian ethical schemata, contrasted with ethical natural law thinking that is rooted in universal ideas of natural justice available to every rational human being, there are ultimately no moral absolutes – even the deliberate taking of innocent human life can be justified to achieve a greater practical end. This philosophical clash, I argue, can provide ethical tinder for MI in the mind of a moral actor.

## Ethics, Morals, and Psychology: An Awkward Tangle

Since Shay and Litz offered their initial definitions of MI (above), much subsequent reflection on the conceptual formulation of MI has focussed on disentangling its connection with post-traumatic stress disorder (PTSD). This is unsurprising since its initial identification was proposed by military psychiatrists working with Vietnam veterans who were poorly responding to conventional therapies for PTSD (Koenig & Al Zaben, 2021; Koenig et al., 2020). While it is generally understood by theorists that MI is not a mental disorder or pathology, the majority of research into MI has been conducted by psychologists using tools of investigation proper to their discipline. Routinely embedded in clinical practice associated with PTSD, this approach is significantly influenced by rationalist assumptions (i.e. where reason, not experience, is the basis of certainty in knowledge) about moral formation and degradation (Kubany, 1994; Schalkwijk, 2018). Although the need for interdisciplinary collaboration is acknowledged for advancing conceptual understanding and for restorative initiatives for MI, psychology continues to dominate research in this field (Griffin et al., 2019; Mallard, 2025; Rabin et al., 2023; Williamson, 2025; Williamson et al., 2023, 2025).

MI is consistently shaped by researchers using psychological or pathological language with theoretical assumptions that can demand subservience to the “hegemony of the symptom” (Kinghorn, 2020, p. 644) located in manualised diagnostic approaches to PTSD (APA, 2013). Such research methodology, as Kinghorn further notes, can act as something of a barrier to developing the MI construct along interdisciplinary lines since “it does not significantly challenge psychiatric research methods or presumptions because it is working from within them” (Kinghorn, 2020, p. 655). Definitional development of the MI construct that plots the increasingly severe impact of moral trauma from moral distress to MI and potential “MI disorder” was recently offered by VanderWeele et al. (2025). This fresh terminology however can be considered problematic for many non-psychologist theorists, for although nuanced, the term “disorder” can imply an unduly and overly medicalised approach that may not be helpful for interdisciplinary collaborative approaches; it risks muddying the conceptual waters if it is not adequately emphasised that the term “disorder” applies only to cases of severe and unresolved instances of moral trauma where a person may come to the attention of psychiatry.

## Interdisciplinary Collaboration: A Natural Way Ahead

In a subsequent paper Mattson, VanderWeele, et al. (2025) described the additional work of their multidisciplinary tertiary collaboration (involving practitioners from psychiatry, psychology, public health, and pastoral care), and their proposed and subsequently approved changes to the DSM-5-TR. The American Psychiatric Association (APA) DSM-5-TR Update to the ‘Supplementary Z Code 65.8’ noted the revised coding from ‘Religious and Spiritual Problem’ to the newly classified ‘*Moral, Religious, and Spiritual Problem’*. The APA update noted the spectrum of ‘moral dilemma’, ‘moral distress’, and ‘moral injury’. The overall classification of ‘*Moral Problem*’ was determined to: “…. include experiences that disrupt one’s understanding of right and wrong, or sense of goodness of oneself, others or institutions” (APA, 2025). The purpose of such a code and descriptor was not to serve as a diagnosis per se, but rather as recognition of a supplementary issue that may be impacting a clinical condition. This development of approach opened the way more clearly to draw on the insights of other disciplines and professionals who are involved in the spiritual and pastoral care of patients and clients. Mattson et al. (2025) warn of the dangers of over pathologising MI, potentially blurring professional boundaries between mental healthcare practitioners (e.g. psychologists) and spiritual care providers (e.g. chaplains). This concern chimes with professional ethical codes of practice within psychology that warn of the dangers associated with practitioners adopting the role of a personal and solipsistic moral authority when treating clients (BPS, 2021; Young, 2017).

Multidisciplinary tertiary collaborative approaches, outlined above, offer a promising contribution to interdisciplinary theoretical understandings of MI. It is worth noting, however, that contemporary psychology is not entirely monochrome in its rationalist assumptions. Jonathan Haidt’s Moral Foundations Theory (MFT) (Graham et al., 2013), for example, gives greater weight to the role of social intuition than previous moral development theorists who privileged the function of deliberate reason in making moral judgements (Kohlberg, 1969; Piaget, 1932/1965). Although not named in Haidt’s MFT, with its pluralist cross-cultural areas of moral foundations (Harm / Care; Fairness / Reciprocity; Ingroup Loyalty; Authority / Respect; Purity / Sanctity), Haidt’s insights resonate with underlying ideas of moral conscience found in the disciplines of moral philosophy and moral theology. In Natural Law thinking, for example, moral intuition is linked with the idea of an innate, pre-reflective moral conscience that gives the foundations or internal operating principles for later moral reasoning. It echoes established research findings on patterns of moral cognition in infants and toddlers that exist pre-strategic reasoning – indeed, in the MFT, Haidt styles this phenomenon as the “universal first draft of the moral mind” prior to any cross-cultural influences (Graham et al., 2013, p. 65). Haidt’s MFT insights resonate with the earlier work of sociologist Karl Kluckhohn that I mentioned above in connection with the universal appeal of a natural ethical approach: Kluckhohn identifies a list of six universal values prohibiting murder, stealing, incest, lying and obliging respect for property and parental relationships (Kluckhohn, 1955). This is an area of intersection with psychology that seems promising for balanced interdisciplinary research initiatives that are not tightly tied to rationalist theoretical assumptions and research paradigms.

## Definitional Clarity Seeking Practice

Incorporating natural law ethical thinking into future definitions of MI could make clearer professional and academic boundaries that could assist clinicians and spiritual care practitioners such as chaplains to determine the most appropriate care pathway for clients. Some of the benefits of an interdisciplinary approach to MI arising from clearer philosophical reflection and delineation in practice could be further explored, for example, in these aspects of its cognitive moral architecture:

- Articulating a clearer awareness of what has actually been violated: is it something that is objectively and universally agreed to be wrong (such as murder or theft) or a more subjective feeling of psychological distress or conflicted feelings due to a situation beyond personal control? A Socratic approach that poses the question of “what is right” could help the person to reflect on the internal workings of their own moral conscience in the light of natural justice themes.
- Conscience violation could be reframed as a normal reaction to injustice or to the violation of *Themis*. Theologians sometimes frame this as appropriate “righteous anger” that scopes a process of healing and restoration by reference to an objective ethical framework and appeal to a benign, higher moral authority.
- Deconstruction of a moral act along casuist approaches^2^ (scrutinising agency, knowledge, intention, and culpability) can help identify what are reasonable moral expectations for an individual moral actor and to distinguish between what may be *objectively wrong* but not *personally culpable*. Moral act deconstruction in light of objective moral norms can also help someone to distinguish between when feelings of guilt and shame are appropriate reactions to a sense of personal wrongdoing – and when they are mistaken errors in thinking. It can also help a moral actor to distinguish between personal and structural or systemic moral failures, with different degrees of attributable moral culpability.
- Clarity regarding if and how an objective, external ethical framework has been violated, for example, transgression of the Golden Rule that is rooted in the Judaeo-Christian 10 Commandments. This insight may suggest a discernible link to arts-based repair options. Religious practices of penance – confession, contrition, atonement, and forgiveness – may offer ways of re-calibrating what looks like “right action” in the mind of a troubled person who is struggling to move beyond destructive feelings of self-loathing. The use of penitential and community-based ritual could be especially helpful in the search for peace of mind and quietening of conscience (Antal et al., 2022; Carey et al., 2023).

## Definitional Clarity in Practice

Definitional clarity, rooted in natural law ethical thinking, could assist clinicians and other professional practitioners to design and pilot referral practices grounded in education and training. This approach would help clarify the nature of presenting issues in connection with possible MI and to consider collaborative intervention strategies. The practice is not new. In healthcare and military contexts, for example, the professional expertise of a multidisciplinary pastoral team is routinely involved in the care of patients and clients. With a sharper philosophical definition of MI, a potential path may emerge for clinicians to acknowledge and integrate natural ethical insights into interdisciplinary practice without the fear of professional boundary compromise.

Natural ethical thinking proposes not a single, unified moral *code* but rather universal, transferable and culturally adaptable ethical *principles* that are rooted in themes of natural justice and operated by the moral conscience in the mind of a moral actor. A natural framework for moral evaluation in a clinical setting could assist the therapist in helping a client explore their conscience-guided assessment of subjective morally injurious feelings by reference to objective principles of natural justice. This process could also enable the client to corroborate or validate their MI. Witnessing the result of an act of inhumanity on the battlefield – say the desecration of enemy dead bodies by a comrade in violation of *jus in bello* – could provoke intense visceral feelings of shame and guilt proper to MI, but the lines of culpability are not initially clear. A natural casuist evaluation of the context could offer hope for healing and moral repair in such situations, by simply noting that some moral actions are objectively morally wrong, even if they are not personally culpable – recognising that the moral conscience has a role in their evaluation. Tools of casuistry found in moral theology (applying moral principles to an individual *case* in context: e.g. was it a free act or was it pressured by external stressors?) can help a person deconstruct a moral act to distinguish what is personally culpable and what is just “wrong”. Soldiers who experience feelings of betrayal by being placed in morally equivocal combat missions by their political masters, questioning stated *jus ad bellum* justifications (Fiala, 2017), may benefit from a casuist element in repair initiatives to understand that MI is not only about “what I do” but also about “what is done to me”. Natural ethical thinking favours a Socratic questioning approach that already works well within some clinical settings (Clarke & Egan, 2015; Law & Allan, 2008). This approach does not *tell* the client what is right and wrong but *invites* him or her to reflect, using their moral conscience, on the actions that are troubling them in the light of natural justice.

### Practical Approaches

The Australian Pastoral Narrative Disclosure (PND) intervention for MI is an example of a well-reviewed chaplain led programme that is built upon interdisciplinary collaboration and trust (Carey et al., 2023). PND uses a comprehensive, integrated humanities-based definition of MI developed in collaboration with the Australian Defence Force (ADF, 2021), that conceptualises MI as “…a trauma related syndrome caused by the physical, psychological, social, and spiritual impact of grievous moral transgressions, or violations, of an individual’s deeply held moral beliefs and/or ethical standards…” (Carey et al., 2023). The elements of this definition sit comfortably with Natural Law thinking that offers  ethical finesse and conceptual clarity to a wider group of affected people than just military veterans (Carey et al., 2025). PND starts in its *Rapport* phase with an established interdisciplinary diagnostic assessment (Moral Injury Events Scale (MIES), Nash et al., 2013) that functions as an initial filter to help the practitioner to establish the nature of the presenting issues and makes a judgement on the level of cross professional referral and intervention that is considered most appropriate. The PND approach moves the degree of professional collaboration from a multidisciplinary to an interdisciplinary operating basis and offers potentially better outcomes for the client that are not siloed in any single definitional or intervention pathway.

A different approach is adopted by another intervention strategy, Litz and Walker’s (2025) *Social-Functional Model of the Treatment of Moral Injury* that is aimed firmly at clinicians and clinical practice. Although treatment strategies offered by this model recognise the value of interdisciplinary collaboration, MI is conceptualised as a “potentially functionally impairing dimensional clinical problem” (p. 259). Litz and Walker’s Social-Functional Model conceptualises MI as distinct from moral distress by its clinical manifestation. The model chimes with the revision of the Z-Codes noted in the DSM-5-TR Update and the floating of the idea of *Moral Injury Disorder* by VanderWeele et al. (2025), and it seeks to add further definitional clarity and precision as the construct develops. Litz and Walker’s (2025) revised definition may, however, have the unintended effect of re-pathologising the term “Moral Injury” in wider interdisciplinary discourse, making Natural ethical thinking sit more awkwardly in the theoretical conversation.

Prior to the changes of the DSM-5-TR Update, and at the other end of the developing MI spectrum (i.e. distinct from a medical approach), cultural anthropologist Tine Molendijk (2022, p. 1) warns of the dangers of *concept creep* or the “expansion of the concept beyond a meaningful scope”. Molendijk’s concern, while initially arising from the danger of a simplistic or reductionist understanding of PTSD, also draws attention to the “enthusiastic uptake” of the language of MI by politically or ideologically motivated third parties, creating conceptual opacity or confusion for MI (ter Heide, 2020). Such conceptual annexation “says more about the moral beliefs of the promoters” than about the phenomenon itself (Molendijk, 2022, p. 2). Lack of philosophical precision and scrutiny of the MI concept may also have the unintended consequence of reducing a naturally occurring manifestation of an affronted, sound moral conscience to a tool of narrow ideological activism, rather than offering an intelligible, universal and objective conceptual framework demanded by any robust ethical theory (Koterski, 2002). A philosophically sharper definition of MI, rooted in natural law ethical thinking, could preserve the concept from theoretical drift by delineating clearer ethical boundaries consistent with its origins in natural justice.

## Morality: A Contested Framework

In philosophical terms, the specifically moral aspect of the MI construct has been historically under-conceptualised in the academic literature (Molendijk, 2018). More recently, however, there has been growing interest in exploring underlying and uniting philosophical approaches and themes that can situate it in wider moral and theological philosophical discourse (Atuel et al., 2021; Farnsworth et al., 2019; Frankfurt & Coady, 2019; Hodgson & Carey, 2017; Papadopoulos, 2020). In their initial paper setting out the building blocks of the MI theory, Litz et al. (2009, pp. 699—701) work within a psychologist’s account of morality, where an individual’s moral thinking is presented as a product of human developmental or shaping influences found in “personal and shared familial, cultural, societal, and legal rules for social behaviour, either tacit or explicit”. Associated manifestations of moral transgression – often expressed in themes of guilt and shame – are typically framed as distorted or irrational cognitions by clinicians (Kubany, 1994). Some of this view finds its roots in the evolutionary psychology work of Freud, and his disciples whose psychoanalytical methodology frames morality and its unconscious influences in terms behaviours useful to society and culture. There appears little awareness that morality might be rooted in anything metaphysical or transcendent. This rationalist approach to morality lodges philosophically in a “descriptive” or relativist framework of understanding (Hare, 1952, 1963), positioned in opposition to a “normative” or code-related understanding of morality that finds in roots in ancient ideas of natural rationality, typically represented by neo-Aristotelian philosophers (Anscombe, 1958; MacIntyre, 2013; Williams, 1985). Gert and Gert (2025) offer an accessible overview of this “is / ought” continuing debate in wider moral philosophical discourse.

Similarly, the moral conscience, in a rationalist account of the mind, is relegated to a function of the Freudian Superego that arises and is replicated in a child’s developmental responses to its parents’ approval and punishment practices (Boag, 2014). This approach to morality can foster a reductive, overly subjective view of MI theory that fails adequately to account for complex social and political influences that assault the conscience (Scandlyn & Hautzinger, 2014; Shay, 2002).

In a clinical setting, there is a need to protect and foster the safe, trusting, and nonjudgemental partnership or alliance between client and mental health professional. Promotion of natural law thinking need not undermine or weaken this professional relationship. It does not suggest that therapists should assume a certain moral approach when interacting with clients. Natural thinking, with its roots in ideas of natural justice, can offer insights into how a client forms moral judgements that might assist the therapist better to tailor intervention strategies. This interdisciplinary approach is not the same as a therapist assuming the role of a moral authority (Farnsworth et al., 2019) – rather it allows for a richer understanding of how moral sensibilities and judgements are formed and evaluated by the moral conscience in the human person. It also suggests to the therapist when the tools of other allied professionals may be helpful for the clients’ repair and restoration. A person of faith whose moral conscience convicts them of sinful behaviour, for example, may benefit from referral to a priest or chaplain for spiritual support as an element of the therapeutic process. This collaborative approach is advocated in some interdisciplinary repair interventions such as Pastoral Narrative Disclosure (PND) (Carey et al., 2023) and Litz and Walker’s (2025) Social-Functional Model.

## Natural Ethical Thinking

Unpersuaded by a purely psychology-driven lens, however, I propose natural law ethical thinking, rooted in unwritten ideas of natural justice, as a common framework that can help to lay open the MI construct by recognising and understanding the multiple personal and structural domains that can lead to moral dilemmas or distress and, eventually, to MI (Carey et al., 2023). This approach has the potential, I believe, to dislodge the developing MI construct from any single academic stable—and to commend its wider application to all domains of the human person by using native human rationality to identify more clearly the demands and transgressions of natural justice in moral actions. Natural ethical thinking, I argue, offers MI theory a stronger philosophical underpinning for its wider interdisciplinary abstraction and inferential application in ways unavailable to competing ethical frameworks such as Deontology or Consequentialism. I say this in recognition that ethical theories rooted in anything other than human nature – such as Utilitarianism – rely ultimately on the deductive exercise of power to compel rather than inductively reflecting on the ethical obligations of the nature of things that is a critical methodological approach of natural law thinking. This understanding of the nature of evidence that sits uncomfortably with scientific empirical assumptions reflects one of the major differences between the two main divisions in research paradigms, quantitative, and qualitative. Attempts to evaluate one paradigm by the tools of the other are doomed to a philosophically non-consummate stalemate (Boyatzis, 1998; Braun and Clarke, 2022).

While acknowledging well-established objections to ethical natural law theory that tend to centre on issues of what constitutes a “good” and concomitant rejection of Aristotle’s central claim of a natural *telos* or a final end or purpose, more recent natural law theorists have sought to be more specific about characteristics of what a “good” might look like, referencing themes located in natural justice (Finnis, 2011; Grisez & Shaw, 1991). This approach, rooted in moral obligations arising from common human nature, is better able to refute serious objections of religious, cultural and moral relativism. Koterski (2002) and MacIntyre (1998) offer accessible and balanced commentaries on developments in natural law thinking and its relationship with later ethical theories. Natural ethical thinking, I nevertheless urge, offers a firmer philosophical foundation on which to scaffold aspirations of trustworthy inference for the application of MI theory more widely. The obliging and persuasive claim of natural ethical thinking, I further urge, is rooted in its ability to appeal to every human being by harnessing the unique characteristic of shared human nature – its rationality.

## *What is Right*: An Ancient Underpinning

Foundational themes of the MI construct consist in a challenge to one’s sense of “what is right” – framed as an assault on the moral conscience or the moral compass that forms the seat of moral authority in the human person. There is little discussion in the academic literature, reflected in the current and still provisional definitions of MI, about what constitutes the basis for “deeply held moral beliefs and ethical standards” (Jamieson et al., 2020; VanderWeele et al., 2025, p. 2). The contested normative/descriptive philosophical approaches to morality, discussed above, frame the conversation and beg the question: is it satisfactory to surrender this central and controlling idea of “what is right” to subjective intuition – or can we look for higher moral themes relating to ethics and moral codes that direct or oblige human behaviour more universally? While some of the theoretical tools of psychology attempt an account of “morality” – clinically, often in connection with disordered cognitions arising from guilt and shame (Kubany, 1994; Kubany & Watson, 2003) – psychology, it seems, has little to say about the “ethics” that give rise to them. It is other disciplines such as moral philosophy and theology that now demand to be given conceptual room to breathe in this complex, ambiguous, and contested developmental space (Kelle, 2020; Kinghorn, 2012, 2020; Koenig et al., 2017; Mallard, 2025; Powers, 2019).

## *Themis:* A Higher Law for Humanity

In his seminal works that laid the foundations for MI thinking, Shay (1994, 2002) represents and reframes the Homeric epic story of the Greek warrior Achilles, who recoils at his experience of the betrayal of “what is right” (*Themis*) by his commander, Agamemnon, during the Trojan War, shunting him into a doom-spiral of destruction and character disintegration. The ensuing sense of moral pollution or *miasma* leads Achilles into a range of deleterious biological, psychic, social, and spiritual manifestations that give rise to the MI construct  (Carey & Hodgson, 2018; Jamieson et al., 2020; Koenig et al., 2017).

Shay identifies the Greek philosophical idea of *Themis* – “what is right” – to frame the behavioural parameters whose transgression is morally troubling to a human person with a soundly intact moral conscience. *Themis* is a persistent theme within ancient Greek literature. Sometimes personified as the Titan goddess associated with the Delphic Oracle, the demands of *Themis* shape and regulate the moral order of things. *Themis* functions as an external pedagogue: a metaphysical reference point or ethical archetype for mortals and their societies. *Themis* is closely associated with unwritten ideas of natural justice – the universally obliging sense of fairness and equity in giving each person what is his or her due that arises from shared human nature.

I return repeatedly to Shay in my argument since he first identified the ancient and enduring roots of MI. In some way, all subsequent theories rest on Shay’s insights. Although Shay has a background in psychiatry, he is able to ask broader questions about the moral origins of MI (in terms of definition) that other MI theorists either avoid or frame using the language and theories of psychology. Shay appeals to ancient and enduring ethical themes rooted in human nature that have resonances for definitions in multiple academic domains and not just medicine or psychology. Later theories seem to start with *what* the effects of MI are on a person – rather than interrogating *why* it should be so (Jinkerson, 2016; Koenig et al., 2019; Litz & Walker, 2025; Litz et al., 2022; VanderWeele et al., 2025). In this way, the symptom can appear of more interest than the cause. Because of his insistence on the causal link between a violation of *Themis* (what is right) and MI, Shay offers a philosophically sharper, clearer, and logical line between cause and effect that other theorists have hesitated to draw. I argue that natural law thinking makes this line clearer in ways that could help future definitions of MI appeal to a wider audience in society as well as in academia.

### Natural Justice, Natural Law, Right Reason

In Greek tragedy, Sophocles (496–406 B.C.) presents the availability of recourse to an undeniable though unwritten *higher law*—the law of Zeus, also rooted in ideas of natural justice—that trumps any positive or codified law and that must be observed in order to *do what is right*, even if this leads to unbearable personal cost or even destruction, as for virtuous and dutiful Antigone (Sophocles, 1984). Appeal to a higher law is a key element in Natural Law thinking, prominent in Greek and Roman Stoic philosophy and jurisprudence. In Natural Law’s first written formulation, the Roman philosopher and statesman Cicero (106–43 B.C.), consolidating Stoic philosophy and political thinking, urges “right reason in accord with nature” as a firm foundation for ethics, observing that:

“There is a true law, right reason in accord with nature; it is of universal application, unchanging and everlasting…. It is wrong to abrogate this law and it cannot be annulled…. There is one law, eternal, and unchangeable, binding at all times upon all peoples; and there will be, as it were, one common master and ruler of men, God, who is the author of this law, its interpreter and sponsor” [De re publica, III.22] (Cicero, 1998).

In Cicero’s formative natural ethical thinking, earlier Greek ideas of *mythos* (didactic or pedagogic stories) mature into later Roman ideas of *nomos* or *ius* (law), giving a firmer, though still unwritten, basis for morality or behaviour that constitutes a pivotal underpinning to evolving Western ethical thought. “Right reason” is here conceived of as human rationality working well to identify the true or proper natures of things, and, flowing from this, the norms associated with the end or telos intrinsic to these natures. In natural law theory, right reason is framed as a moral capability or power that enables human beings to discern what is right from what is wrong, what is good from what is bad. Foundational and enduring ethical formulae such as the Judaeo-Christian 10 Commandments and the Golden Rule (rooting Kant’s Categorical Imperative) give more detailed, thematic, expression to this thinking.

### Moral Order

In her synoptic comparison of Dante’s moral themes and the MI literature, Suurmond (2026) identifies the need for a commonly agreed moral order to secure justice and prevent violence in society (theme #12). Tyranny, as Dante notes, overturns the moral order – sowing the seeds for MI. In Dante’s thinking, there is a *need for a coherent moral order or ethical framework to guide human decision making* that has both personal and societal value. Dante not only alludes to Marcus Aurelius’s famous aphorism on the workings of a swarm of bees (quoted below) but also roots his moral philosophy in the thinking of St Thomas Aquinas on the Natural Law. Suurmond further notes that modern secular society has replaced much of the moral vocabulary necessary for moral discernment and evaluation with value-free or morally neutral language. In preferring terms such as “traits” or “transgression” over “good / evil” or “virtue/vice,” for example, Suurmond contends that terms that might be clinically useful may have the knock-on effect of not only impoverishing critical moral intelligence but also of limiting ability to identify injustice. Against this background, a natural law ethical approach (in conjunction with Socratic questioning) could offer a way for the person to morally evaluate their actions – thereby suggesting ways in which the therapist could help the person understand and come to terms with their actions. This awareness could be a starting point for multidisciplinary referral (e.g. a chaplain or even a lawyer).

## Natural Law Ethical Thinking Endures

Also known by other names across the centuries (such as *human dignity, natural, or human rights*), natural law thinking has resurfaced thematically, though sometimes tangentially, in nearly all Western ethical theories. It arises in human rights principles embodied in International Humanitarian Law, where appeal is made to a “supra-positive” law – a higher law of justice that trumps all others. After World War II, the justices at the Nuremberg trials of Nazi war crimes, for example, used recourse to the idea of a suprapositive or higher law arising from natural law thinking to convict Nazi war criminals of “crimes against humanity” since they were unable to convict under the national positive laws of any of the participating nations (Rommen, 1959). Similarly, the incarcerated 1960s civil rights campaigner Dr Martin Luther King Jr wrote his *Letter from the Birmingham Jail* (King, 1964), making his case against contemporary racial discrimination *positive* laws by appealing to the idea of a higher *supra-positive* law rooted in ideas of natural justice that ground natural law thinking in human rationality. King references the natural law theorist, St Thomas Aquinas (c.1225–74), in making his argument (Aquinas, 1952).

These examples offer insight into the philosophical strength of a natural approach to ethics that is not bound by cultural, political, or religious theoretical assumptions – yet which is capable of subsisting, to some degree, within ethical theories that are more explicitly indebted to ideas shaping the main Western ethical theories referenced earlier. Natural thinking asserts that objective and morally obliging ethical operating principles may be discovered through a chronic process of human rationality reflecting on human nature. Herein, I argue, is the natural approach’s strength to persuade and to endure.

## *Themis* and MI: An Operating Dynamic

Shay notes that ideas of *Themis* touch the very structures of social interaction such as convention, behavioural norms, ethics, and commonly agreed social values. *Themis* lays the ethical foundations or social operating principles of “what is right”. Betrayal of *Themis,* Shay notices, is personally corrosive and “particularly destructive to a sense of continuity of value in ideals, ambitions, things, and activities. When some major ideals have been betrayed, the trustworthiness of every ideal or activity may be called into question” (Shay, 1994, p. 178). In his subsequent volume *Odysseus in America*, Shay further unpacks the sense of a betrayal of natural justice – *Themis* ambushed, so to speak – as experienced by homecoming Vietnam veterans whose natural capacity for social trust is crushed by their lived experience, constituting further personal and societal tinder for MI (Shay, 2002).

Because of its direct link to ideas of natural justice, *Themis*, I argue, is the universal, scaffolding and controlling idea of “what is right” whose betrayal makes the concept of MI possible—and whose restoration in the minds of the morally injured seeks a track to healing that resists containment in any one academic or professional approach. Shay’s anchoring references to the idea of *Themis* in his initial thinking around the concept of MI are an example of *ressourcement*, a theological device of returning again to the sources or origins of a phenomenon or concept to give fresh insight and application to the present-day context (Pecklers, 2011). As the MI concept ripens as an interdisciplinary project, the insights and toolkit of moral philosophy and moral theology can have an increasingly important contribution not only to its theoretical formulation but also for rich ideas informing arts-based repair interventions (Brock & Lettini, 2012; Carey et al., 2023; Fiala, 2017; Papadopoulos, 2020).

## A Plea for Philosophical Scrutiny of the MI Construct

Individual beliefs about what constitutes “what is right” can vary, drawing upon diverse influences such as culture and religious formation. Such beliefs may be subjective, contested, and intensely personal – and it is not my intention here to attempt to review the long and complex history of Western ethical thinking. However, I do argue that to preserve the MI construct from a subjective and intuitionist cul-de-sac of ethical thinking, there needs to be clearer understanding and agreement around the ethical operating principles that constitute “what is right” and ways in which they can be transgressed.

This line of argument begs the question: is there moral equivalence between all ethical frameworks that resists moral evaluation—or are there some non-negotiable ethical principles, such as the universal prohibition of intentionally taking innocent human life, that demand universal allegiance? If so, where are they located and who is the arbiter? Is violation of subjective moral intuition, independent of a wider ethical framework or world view, a sufficient spark to ignite a MI? Without clearer rooting of the moral conscience in themes of natural justice, current proposed definitions of MI could be understood as relying so heavily on subjective intuition that they can claim limited ability to persuade. The stoic Roman emperor Marcus Aurelius (121–180 A.D.) pithily captured the tension present in the need for alignment between an individual moral actor and ethical metaphysics when he ironically observed “that which does not benefit the swarm, does not benefit the bee” (Aurelius, 1983, p. 60). Natural law ethical thinking offers a moral mooring, I argue, precisely because it does not depend on subjective ideology, power, or compulsion to persuade a moral actor to “do the right thing”; it appeals instead to the unique characteristic activity of humanity, our native *rationality* chronically reflecting on the ethical nature of things and their internal operating principles ordered by enduring ideas of natural justice.

I offer the above comments not only to plant a Socratic *reductio ad absurdum* in the reader’s mind but also to argue that, if the MI construct is to gain wider acceptance in academia, and in society generally, as a robust and serious ethical theory, it needs to be compatible with principles that are required for any ethical thinking, capable of withstanding closer philosophical scrutiny. In short, by employing natural law thinking, the MI construct can withstand thematic scrutiny seeking: *Intelligibility* (something able to be known plainly and simply, without the need for advanced education); *Objectivity* (advancing arguments that anyone of an open mind and goodwill finds compelling, arising not merely from personal intuition or subjective preference) and *Universality* (something that applies to every human being) (Koterski, 2002). Natural law ethical thinking, I therefore argue, offers a persuasive philosophical framework for the MI construct to flourish in—and to defend itself from multi-sided and subjective concept drift and ideological annexation (Molendijk, 2022; ter Heide, 2020).

## Ethics, Morals, and Conscience: A Conceptual Dynamic

I have argued so far for a clearer conceptual line that connects ethics (a system of thematic operating principles) and morals (those principles in action). If, as I contend, philosophical themes found in natural justice (*Themis*) and natural law can scaffold an intelligible, objective and universal system of ethics against which moral actions can be evaluated thematically, what is the practical mechanism available to a moral actor for connecting its transgression with a sense of MI?

Shay does not unpack his understanding of the working of the moral conscience in his *Achilles* and *Odysseus* books – though he does briefly name it. In a later paper that forms his working definition of MI (Shay, 2014), there is no specific mention of the term “moral conscience”. This is not to say that Shay is unfamiliar with or dismissive of the idea of the moral conscience’s role in evaluating and directing a moral action. Rather, I suspect, he is tacitly content that the idea of conscience sits quietly in the undergrowth of “what is right” informing deeply held moral sensibility. Uninterrogated as to its mode of operation by Shay, it awaits richer complementary lenses of investigation that moral philosophy and theology can offer as the conceptual wheel turns and matures (Kelle, 2020; Kinghorn, 2012; Papadopoulos, 2020; Powers, 2019).

The role of the moral conscience in the formation of moral distress and MI is prominent in Brock and Lettini’s (2012) book, *Soul Repair*. Here are echoes of Rousseau’s (1712–1778) intuitive framing of conscience in his 1755 work *Emile*, as a witness to an *innate principle of justice and virtue* (Rousseau, 1911). Using a natural law lens, Rousseau fathoms that “conscience persists in following the order of nature in spite of all the laws of man”. Insights of philosophy, theology, poetry, and the social sciences help to position MI more clearly as an assault on the moral conscience, identifying the need for “soul repair” for those touched by it. *Soul Repair* strikingly starts with a quotation from a US Army chaplain (Rev Keizer) that reflectively frames the seriousness of conscience violation in terms of “moral suicide” (Brock & Lettini, 2012, p. xi).

## Conscience: A Spiritual Foundation

Later writers have adverted to the central role of the moral conscience in the MI construct (Kelle, 2020; Panchuk & Thirsk, 2021; Yanko, 2025), though with little philosophical comment on the conceptual glue that links the ethics, morals, and conscience dynamic that I here urge is important. Brock and Lettini’s (2012) framing of MI as an *injury to the soul* is significant because it repositions MI away from medicine into the metaphysical domain of the spiritual, the framing territory of the moral conscience within the human person, the moral actor. There are echoes in this approach with US Iraq Veteran Tyler Bourdeau’s earlier comment that by *spiritually rescuing* the MI construct from medicine, it offers the possibility of “transform[ing] ‘patients’ back into citizens and ‘diagnoses’ into dialogue” (Boudreau, 2011, p. 750). For clarity, I understand and use the term ‘spiritual’ in the sense adopted by the World Health Organisation as “that which gives meaning and purpose to one’s life and connectedness to the significant or sacred”, rather than in a primarily religious sense (Brémault-Phillips et al., 2015, p. 477; Carey & Cohen, 2015).

Brock returns to consider the role of the moral conscience in her chapter exploring moral conscience and ritual in Papadopoulos’s multidisciplinary collection of essays, observing that, through the use of empathy:

“Moral conscience is not just inner subjective intent, i.e. the desire and choice do the right thing, but a complex, interactive, and fallible system of action, intention, and evaluation that constitutes good character”. (Papadopoulos, 2020, p. 38)

Brock seems to resonate with Aristotle (384 – 322 B.C.), for whom, as presented in his *Nicomachean Ethics*, the personal moral excellences (*arete*) that form good character are firmly rooted in ideas of natural justice (Aristotle, 2013). In a clearer philosophical approach, Atuel et al (2021) explore Aristotle’s linkage of character formation and its disruption by morally troubling experiences to comment on the transgressive aetiology of MI, though without explicitly referencing the function of the moral conscience in the process. Aristotle’s moral virtue of prudence (*phronesis*) – the personal excellence of knowing what to do for the best outcome of a moral act by deliberating realistically and then choosing it in a timely way—is similar, though not identical, with what would later be known as conscience. The internal operating principles of both prudence and conscience, however, arise in the native human power of rationality. This view is distinct from rationalist theories of conscience that are typically advanced by the discipline of psychology, often linked with ideas of guilt and shame arising from childhood influences and social or cultural pressures. In this rationalist approach, empathy is identified as one element that influences the formation of the conscience that arise from – or even co-ordinate – the powers of self-conscious emotions and moral reasoning in the human psyche (Schalkwijk, 2018).

Returning to Brock’s (2020) observation on the link between conscience and character, empathy alone, however, does not fully account for how conscience arises, develops, operates and even fails. It requires further philosophical comment. If the moral conscience is the governing factor in how connections and evaluations are made and witnessed between ethics and morals, or principles and practices, it must have recourse to a framework more robust and stable than human intuition alone. Conscience’s necessarily external, metaphysical, and spiritual nature was argued by Cardinal Newman (1801–1890) who characterised the moral conscience as:

“A voice within us, which assures us that there is something higher than earth. We cannot analyse, define, contemplate what it is that thus whispers to us. It has no shape or material form” (Strange, 2009, p. 130).

In arguing such, Newman reaches back into Greek ideas of *Themis* that Shay built his initial definition of MI on. This understanding of moral conscience also references ideas of a *higher law*, heartbreakingly articulated by Sophocles’ *Antigone* and reaffirmed by Cicero’s Natural Law formulation, above. It offers a mechanism for a moral actor to avoid objections of subjectivity and moral relativism that arise from purely psychological, social, or anthropological understandings of the roots of the moral conscience that are philosophically indebted to post-Enlightenment ideas arising from *rationalism*—rather than from enduring, ancient ideas of native human *rationality* that shape natural law ethical thinking. In short, the moral conscience offers an enabling connection—a *recapitulation* even—between the subjective (morals) and the objective (ethics) (Melina, 2026).

## Limitations and Future Lines of Research

Sitting firmly within a qualitative and interpretivist research paradigm, this paper differs in approach from the character of most mainstream Moral Injury scholarship. The philosophical approach used to add conceptual clarity to evolving definitions of MI does not lend itself to scientific empirical methodology – even when using mixed-methods approaches. In this qualitative paradigm, issues of empirical rigour, validity, and reliability are evaluated using established alternative criteria. Its appeal to some clinicians working within a different research paradigm may therefore be limited. Complementary lenses and investigative tools grounded in qualitative research methodology, however, offer a richer foundation for interdisciplinary collaboration in exploring future definitions of MI.

This paper’s argumentation relies on natural law thinking, rooted in natural justice, as a framework to add philosophical scrutiny and precision to the emerging concept of MI. Natural ethical thinking remains contested as an ethical theory—and a natural approach cannot address all ethical questions—but I have argued that, in comparison with rival Western ethical theories, natural law theory offers a distinctively objective, intelligible and universal way to understand MI since it is uniquely grounded in commonly available human nature and experience. Given this constraint, a natural approach to ethical theory offers conceptual shared ground that can offer an accessible philosophical lens to multi-dimensional contributions to developing MI theory.

Looking ahead, future research could be fruitful in two main areas:

1. It could continue to develop the interdisciplinary academic foundations of a philosophical scaffold to shape definitional clarity of MI as a discrete ethical theory. Contributions of psychology, theology, philosophy, literature/poetry, social sciences could all enrich and advance conceptual understanding of MI.
2. A natural law lens could be used to enhance and complement clinical approaches with an interdisciplinary element of diagnosis and subsequent community-based repair strategies. Collaborative development could build on existing research such as PND (Carey et al., 2023, 2025)  and other reported mental health/chaplaincy initiatives (Jones et al., 2022; Koenig et al., 2023). This pathway would also reference recent advances in psychiatry found in the latestrevision of the DSM-5-TR updated Z-codes that added *moral* issues to the existing spiritual andreligious category, opening the door to wider cross-disciplinary collaboration (Mattson, et al, 2025).

## Where Now?

I began this paper by articulating an *aporia* – a developmental pause or question favoured by Socrates as a stimulus for discussion—arising from a philosophical lens taken to current working definitions of MI. The phrase “what is right” is a key building block of the MI construct, having its roots in the Greek philosophical notion of *Themis,* as proposed by its architect Jonathan Shay (1994). Natural law ethical thinking adds a robust and enduring philosophical underpinning to the universally obliging character of natural justice that *Themis* embodies. Later theorists have built on this philosophical foundation – suggesting ways in which *Themis* might be wounded or even destroyed by MI – yet with little curiosity about an ethical framework that gives rise to the basis on which a moral actor knows and chooses “what is right” and its evaluative mechanism, the moral conscience. I have argued, philosophically, that the term “moral” necessarily begs the question of *which* ethical framework the term references and to ask whether MI could be rooted logically in any or every version of ethics? Further philosophical reflection and comment on this question is required for future conceptual development of the MI construct along interdisciplinary lines.

My argument here is that in order to remain aligned with the ancient and controlling roots of *Themis,* and its persistence in Western ethical thinking that Shay drew attention to in his ethical representation of Homer’s Achilles in the *Iliad*, foundational themes residing in natural justice thinking and ethical natural law theory can add clarity in the quest for conceptual distinction and agreement. Such thinking offers an intersectional way forward for MI theorists in the search for an acceptable, interdisciplinary, and commonly agreed definition of their subject. I further argue that the role of the moral conscience—with its issues of origin, formation, maintenance, and deformation—finds an archetypical ethical reference point in ethical natural law thinking. I make a case for referencing explicitly natural themes of justice and equity in emerging definitions of MI. This approach, I argue, has the potential to offer a robust philosophical foundation to the MI construct that can be demonstrably and reliably intelligible, objective, and universal in character.

Grounding the developing MI construct in natural law thinking can offer not only interdisciplinary collaboration in clinical practice – an approach invited by the recent inclusion in the Z-Codes referencing moral problems in psychological assessment (APA, 2025; DSM-5-TR, Z65.8) – but also in developing further arts and spiritual-based intervention or repair initiatives for those affected by MI, such as meditation or story telling (Fleming, 2021, 2022).

In a similar literary approach to Shay’s initial works (1994; 2002), for example, Suurmond (2026) excavates Dante’s epic *Divine Comedy* to offer a worked example of interdisciplinary collaboration between psychology, philosophy, poetry, and moral theology that offers a broader, humanities focused account of the MI construct that is not tied to one research paradigm. A natural approach to MI, rooted in foundational themes of justice, I argue, has the potential not only to safeguard human rights discourse away from political manipulation or power dynamics but also to restore the concept to philosophical foundations located in shared humanity.

## Footnotes

1 The United Nations Charter of 1945 offers a constitutive account of the core elements of a contemporary understanding and application of classical Just War thinking (United Nations, 1945).

2 Casuistry is the art of applying general ethical principles to individual moral situations or *cases*. It is a method central to penitential theology.
